# Investigation of Crack Propagation Behaviour in Thin-Rim Gears: Experimental Tests and Numerical Simulations

**DOI:** 10.3390/ma16114095

**Published:** 2023-05-31

**Authors:** Haifeng He, Andrea Mura, Taihua Zhang, Heli Liu, Weiping Xu

**Affiliations:** 1 School of Mechanical and Electrical Engineering, Guizhou Normal University, Guiyang 550025, China; haifenghe@gznu.edu.cn (H.H.);; 2 Department of Mechanical and Aerospace Engineering, Politecnico di Torino, 10129 Torino, Italy; 3 Department of Mechanical Engineering, Imperial College London, London SW7 2AZ, UK

**Keywords:** thin-rim gear, extended finite element method, crack path, crack propagation

## Abstract

Thin-rim gears are widely used in industrial fields such as aerospace and electric vehicles due to the advantage of light weight. Yet, the root crack fracture failure of thin-rim gears significantly limits their application and further affects the reliability and safety of high-end equipment. In this work, the root crack propagation behavior of thin-rim gears is experimentally and numerically investigated. The crack initiation position and crack propagation path for different backup ratio gears are simulated using gear finite element (FE) models. The crack initiation position is determined using the maximum gear root stress position. An extended FE method coupled with commercial software ABAQUS is used to simulate the gear root crack propagation. The simulation results are then verified by conducting experimental tests for different backup ratio gears based on a dedicated designed single-tooth bending test device.

## 1. Introduction

Gears are one of the most important components of essential mechanical transmission; requirements for the precision, light weight and anti-fatigue properties of gears are particularly important. Thus, lightweight gears, such as the thin-rim gears, are popular in aerospace and automotive applications (e.g., electric vehicle transmissions) [1]. To achieve weight reduction, the geometry of these gears consists of a web connecting the hub to the rim that stands behind the teeth. However, lightweight gears are prone to crack failures. In particular, if a crack nucleates near the tooth root, it may propagate through the tooth (as in bulk gears) and also in the radial direction, leading to the removal of a large portion of the gear and resulting in catastrophic consequences.

Crack propagation in thin-rim gears has been increasingly investigated from numerical and experimental aspects. Lewicki [2] from NASA is a pioneer focused on the lightweight investigation of steel thin-rim gears used in helicopter or turboprop power transmissions. Based on experimental tests and FE methods, Lewicki et al. [3,4,5] analysed the crack propagation path and obtained the corresponding fatigue life considering the effect of rim thickness, centrifugal load and initial crack propagation position. They found that the longest fatigue crack life emerges in such cases where the backup ratio is neither small nor large. These results were subsequently used for the ultra-safe design of gears [6]. Moriwaki et al. [7] conducted a series of fatigue tests for POM-C plastic spur and helical gears with different rim thicknesses. Experimental results show that the rim thickness changes the root crack growth path, and the fatigue life of plastic gears decreases as the rim thickness decreases. Oda and Miyachika [8] analysed the effects of keyway position on the gear stress and fatigue crack initiation for thin-rim gears based on experimental tests. They found that the position of the keyway should satisfy the condition that the centre line of the keyway coincides with that of the gear tooth. Kahraman et al. [9] investigated the effect of rim thickness on the stress, deformation response and the load sharing of a planetary gear set in automotive transmission applications. They found that the deflections of ring gears should be considered during the gear design. Supported by fractography and metallurgical methods, Xu et al. [10] found that the first crack of a 12 mm modulus wind turbine gear, which was mainly caused by non-metallic inclusions, initiates approximately 3 mm from the groove during the carburization-quenching process. Yan et al. [11] investigated the fatigue crack propagation behaviour using bending fatigue tests with case-carburized and shot-peening gears and found a crack trajectory of around 30° and a relaxation of residual stresses.

With the development of computer science and technology, gear root crack propagation investigation based on numerical simulation is attracting more attention. Coupling linear elastic fracture mechanics (LEFM) with FE code, Zouari et al. [12] simulated the crack propagation path for different backup ratio gears and found that cracks would propagate to the rim when the backup ratio was less than 0.38. Using the extended finite element method, Cura et al. [13,14,15,16] established a series of simulation models for the root crack propagation of gears, considering the effects of rim, web thickness and centrifugal load. Doğan et al. [17] investigated fatigue crack propagation using numerical simulations, obtaining the directions of the cracks for different rim thicknesses, drive side pressure angles and durations. Nandu et al. [18] studied the effect of backup ratio on the fatigue crack behaviour of symmetric and asymmetric spur gears under mixed-mode fracture. The results show that the gear fracture strength increases as the gear rim thickness increases. Kramberger et al. [19,20] studied the fatigue crack of thin-rim gears from a truck gearbox. The crack propagation path, stress intensity factors in the crack tip as well as the fatigue crack life were investigated via a boundary element method (BEM) using LEFM. They assumed that the fatigue crack propagation life for non-strengthened gear rims was longer than that of the gear rim reinforced with webbing. For the aim of predicting the crack propagation path rapidly, a factorial design method coupled with the boundary element were adopted by Lalonde and Guilbault [21], with different gear geometries and initial crack configurations. The established model enabled instant modelling of the crack trajectory in thin-rimmed gears throughout the entire lifetime. Based on the pseudo-evolutionary structural optimization method, Gueye et al. [22] predicted gear crack growth paths with different backup ratios. This method, taking the maximum bending stress as an indicator, enabled the simulation of the crack propagation beyond the fracture mechanics frame. Podrug et al. [23] investigated the effects of moving load on the gear fatigue crack path and fatigue life using the critical plane damage method and fracture mechanics. They found that the crack path for the moving load case was different from that of the conventional gear fatigue pulsating test.

Utilizing the gear fatigue limit diagram and the gear root cyclic stresses, Miyachika et al. [24] evaluated the bending strength of thin-rim gears. Li [25] proposed a 3D FE thin-rim gear model to investigate the gear deformation and stress response. The results show that the gear deformation mainly occurred in the rim and web (70% of deformations) rather than the gear tooth. In addition, the root stress in the thin-rim gear was significantly greater than in the solid gear. To analyse the effect of centrifugal forces on the fatigue life of thin-rim gears, Opalić et al. [26] developed a 2D gear FE model following the plane stress assumption. A strain–life approach considering the mean stress correction was applied for different backup ratios. They assumed that the backup ratio would have a more significant influence on the bending fatigue life compared to the centrifugal force. The uniformly distributed Timoshenko beam theory was applied by Chen et al. [27] to calculate the internal gear mesh stiffness considering the influence of the ring gear rim deformation. Karpat et al. [28] conducted a parameter sensitivity study on the effect of rim thickness on the gear bending stress response and mesh stiffness using FE simulation. They found that these two factors decrease as the rim thickness increases. Lin et al. [29] established a three-dimensional spur gear pair used for a 4LZ-2 combined harvester to determine the tooth root fatigue crack initiation position for the pinon gear based on the bending stress history during the meshing process. Therein, the fatigue life was predicted utilizing the power density method and fracture mechanics. He et al. [30,31] predicted the gear bending fatigue life based on the continuum damage mechanics and fracture mechanics. The estimated fatigue life agreed well with experimental test. Vučković et al. [32] simulated the gear bending fatigue for two gear loading configurations. They assumed that the friction significantly affects the position of first crack initiation in the root area.

In this work, the root crack propagation behaviour of thin-rim gears is investigated through experimental tests and numerical simulations. The crack initiation position and crack propagation path for different backup ratio gears are predicted using FE simulations. The gear experimental tests were performed on classical-shaped thin-rim gears with a dedicated designed single-tooth bending test device.

## 2. Experimental Setup

**Figure 1:**
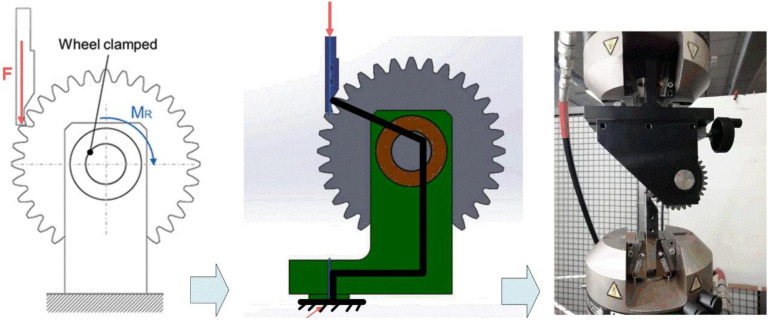
Single-tooth bending test.

The INSTRON servo-hydraulic fatigue testing machine with maximum 100 kN loading force (INSTRON 8801, INSTRON CORPORATION, Boston, MA, USA) is utilized to test the crack propagation path of thin-rim gears. The single tooth loading condition is adopted using a dedicated device (Figure 1). The test gear is clamped on the shaft, the loading punch is fixed through two clamp parts, and a tooth of the test gear is loaded via the punch. The clamping condition of the test gear enables the reproduction of a loading condition very close to the actual working condition: the load is applied on a tooth, and it is transferred to the shaft (Figure 1).

Gear samples with a teeth number of 32 and modulus of 3 mm are used in this study. The gear geometry and material parameters are listed in Table 1. The chemical composition of C45E gear steel material is listed in Table 2. The tensile strength of the gear steel is 745 MPa. To investigate the effect of rim geometry on the gear root crack propagation path, gear samples with different geometry are applied in the gear bending fatigue test and FE simulations. 

**Figure 2:**
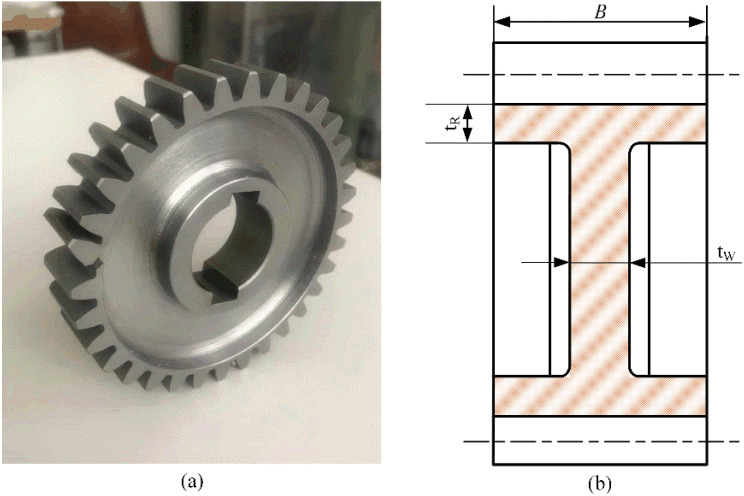
Gear test sample (**a**) and geometry (**b**).

The values of the backup ratio were selected in order to reproduce, according to the references, a “failsafe” breakage ($M_{R}$ = 0.5) and a “catastrophic” breakage ($M_{R}$ = 0.3). The backup ratio $M_{R}$ and the web ratio $M_{W}$ are defined as

(1) $$M_{R}=\frac{t_{R}}{m_{n}}$$

(2) $$M_{W}=\frac{t_{W}}{B}$$

where $t_{R}$ is the rim thickness, $t_{W}$ the web thickness, *b* is the face width and $m_{n}$ is the normal gear modulus, as shown in Figure 2. Using this gear geometry, four configurations ($M_{R}=0.3,0.5,1$ and bulk gear) are selected for simulations. The gear face width is set as a constant and $M_{W}=0.1$.

## 3. FE Simulations

**Figure 3:**
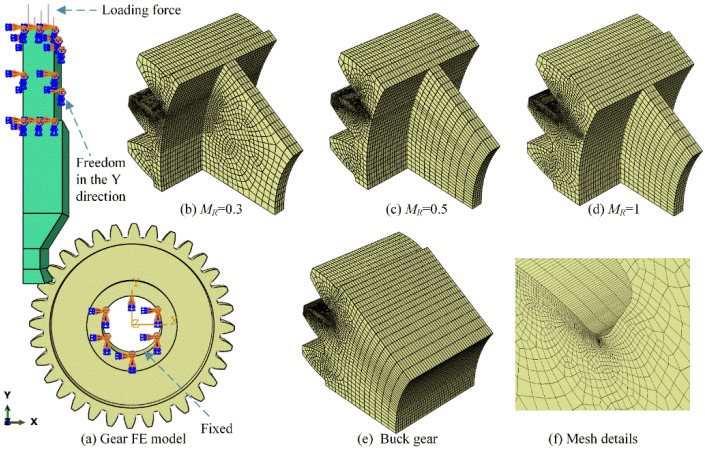
Gear FE model for crack initiation position determination.

The extended finite element method coupled with commercial software ABAQUS is used to simulate the gear root crack propagation. Figure 3a shows the gear bending FE model for crack initiation position determination. The boundary conditions and loading force in the gear FE model are consistent with the experimental test for verification. The gear is fixed by a combined clamp, and the upper punch can move only along the y-axis direction. The loading force of 6000 N is applied on the upper punch and then transforms to the gear surface through the highest point of single tooth contact (HPSTC). Four types of gear with backup ratios (0.3, 0.5, 1 and buck gear) as shown in Figure 3b–e are applied to analyse the effect of rim thickness on gear root crack propagation. An 8-node hexahedron linear reduced integral element C3D8R is used on the gear and the punch parts. Figure 3f shows that a refined mesh is applied in the gear root area to achieve accurate computation. In addition, gradually rougher mesh is used to reduce the simulation time.

**Figure 4:**
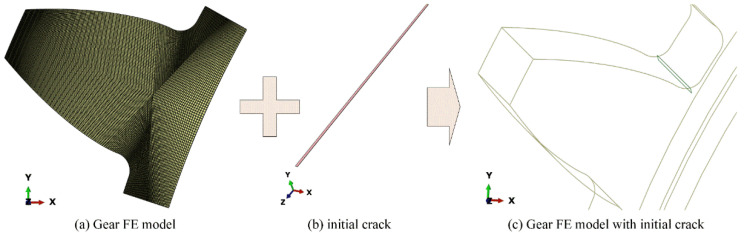
Cracked gear finite model for crack propagation simulation.

Figure 4 shows a cracked gear FE model is used to simulate the gear root crack propagation. A more refined mesh is adopted to ensure the stability of gear crack propagation simulation. Figure 4a shows the detailed mesh of the gear model, where the minimum mesh size is 0.02 mm. Figure 4b shows the initial crack, which is prefabricated on the gear root area. The length of the initial crack is set as 0.2 mm. Figure 4c shows the final gear crack propagation FE model, where the position of initial crack is determined based on the calculated gear bending stress.

## 4. Results and Discussion

### 4.1. Gear Finite Element Model Stress Convergence Verification

Four different element sizes (minimum mesh sizes of 0.2, 0.1, 0.05 and 0.025 mm) are used to determine the bending stress convergence. The gear bending stress is based on the international standard ISO 6336-3 [33]. Following this standard, the gear root bending stress is calculated as

(3) $$\sigma_{\left(ISO\right)}=\frac{F_{t}}{b\cdot m_{n}}\cdot Y_{F}\cdot Y_{S}$$

where $F_{t}$ is the tangential load with the unit of N. $Y_{F}$ and $Y_{S}$ are the geometry factor and stress concentration factor, respectively. $Y_{F}$ and $Y_{S}$ are derived as

(4) $$Y_{F}=\frac{\frac{6h_{Fe}}{m}\cos\alpha_{Fen}}{{\left(\frac{s_{Fn}}{m}\right)}^{2}\cos\alpha_{n}}$$

(5) $$Y_{s}=(1.2+0.13L)q_{s}^{\frac{1}{1.21+\frac{2.3}{L}}}$$

where $S_{\mathrm{FN}}$ and $h_{\mathrm{FE}}$ are the tooth root normal chord and the bending moment arm at the critical section, respectively. $\alpha_{n}$ is the normal pressure angle, and $\alpha_{\mathrm{Fen}}$ is the load direction angle. $\rho_{F}$ is the radius of the root fillet in the critical section. The parameters L and $q_{s}$ are calculated as

(6) $$L=s_{\mathrm{FN}}/h_{\mathrm{Fe}}$$

(7) $$q_{s}=s_{\mathrm{Fn}}/2\rho_{F}$$

**Figure 5:**
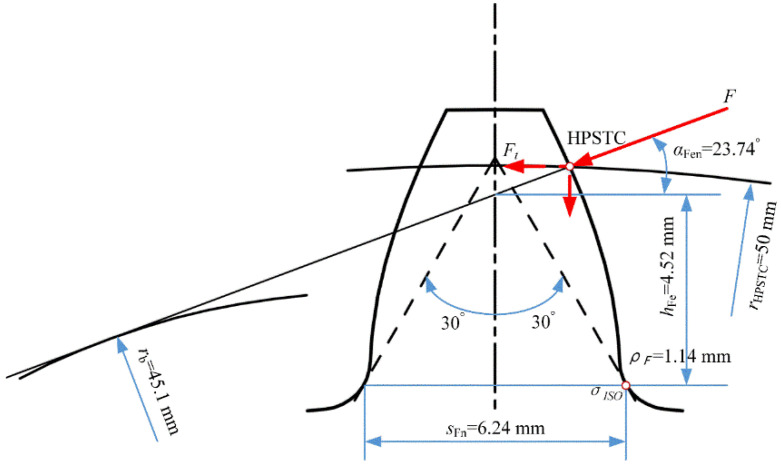
Geometric parameters for bending stress calculation based on ISO 6336.

The detailed values of these geometric parameters for this gear sample are depicted in Figure 5. Accordingly, the geometry factor and stress concentration factor are $Y_{F}=2.04$, $Y_{S}=1.96$. The load force $F_{n}$ of 6000 N applied by the punch is applied on the HPSTC of the tooth surface, and the tangential load force is calculated as

(8) $$F_{t}=F_{n}*\cos(\alpha_{\mathrm{Fen}})$$

**Figure 6:**
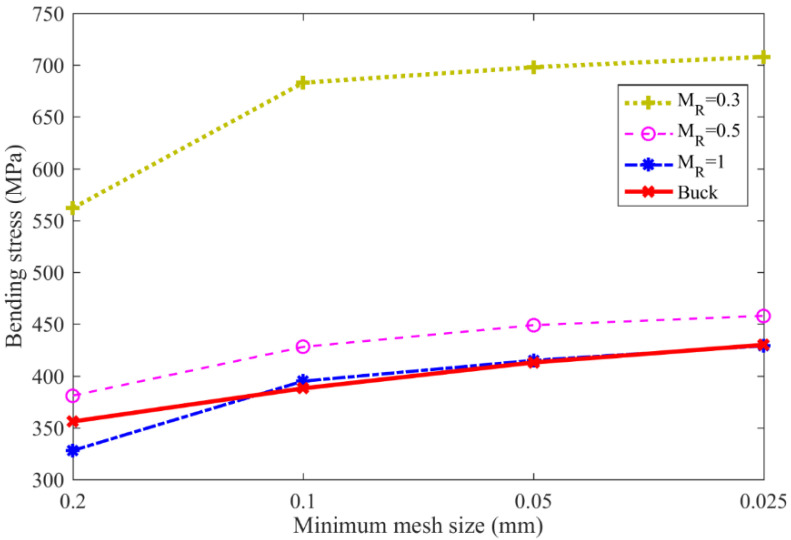
Gear bending stress under different mesh sizes.

Figure 6 and Table 3 show the gear bending stress obtained from the FE simulation and ISO standard. The ISO-based gear bending stress values are kept constant at 365 MPa with different backup ratios, since the effect of rim thickness on gear root stress is neglected in the ISO 6336 standard. However, the bending stress increases as the rim thickness decreases in engineering practice. For example, the bending stress increases dramatically from 429 MPa to 708 MPa as the backup ratio decreases from 1.0 to 0.3. When the backup ratio reaches 1.0, the effect of rim thickness on the gear bending stress is extremely small; however, this effect needs to be considered when the back ratio is less than 0.5.

Figure 6 shows the gear bending stresses obtained with different mesh sizes. The gear bending stress for different backup ratio cases gradually increases as the element mesh size decreases, while bending stress increases more gradually as the mesh size decreases. Specifically, the bending stress for the backup ratio of 0.3 increases from 562 MPa to 683 MPa, where a 21.5% growth is observed when the mesh size decreases from 0.2 mm to 0.1 mm. This value increases from 698 MPa to 708 MPa, with only a 1.4% growth, when the mesh size decreases from 0.05 mm to 0.025 mm. This indicates that when the minimum gear mesh size reaches 0.05 mm, the gear root stress convergences in the gear FE models. Hence, this mesh size is used in the following gear crack propagation simulation.

**Figure 7:**
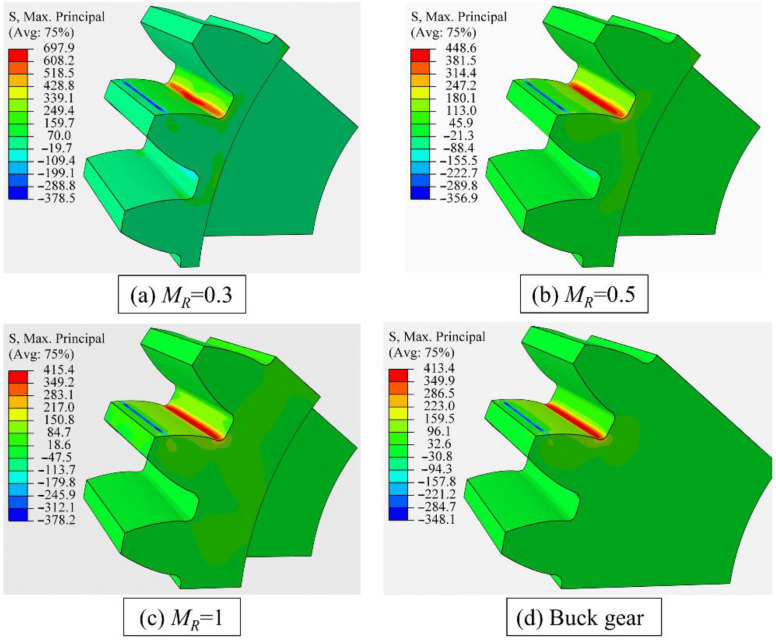
Gear bending stress contours for different backup ratios under element mesh size of 0.05 mm.

Figure 7 shows the gear bending stress distributions for different backup ratios under an element mesh size of 0.05 mm. The shapes of stress contours are similar to each other, even though the maximum gear root stress under the same loading force (F = 6000 N) is quite different due to different rim thicknesses.

### 4.2. Crack Initiation Position

**Figure 8:**
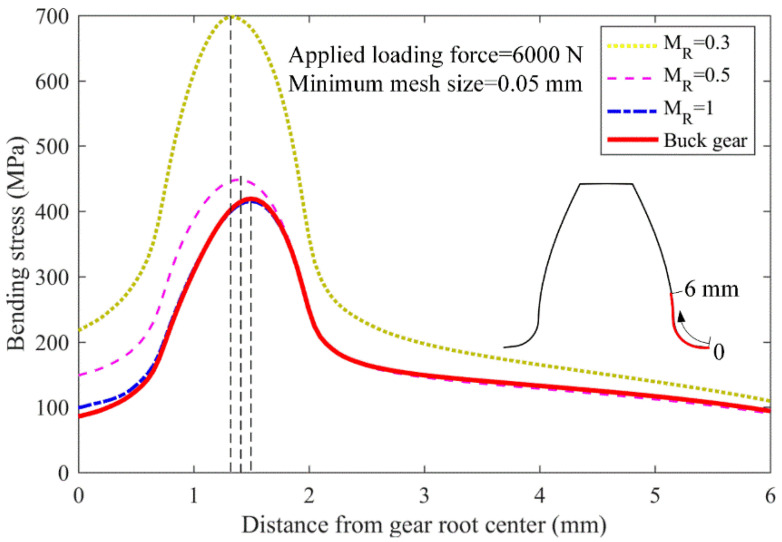
The maximum gear root stress position for different backup ratios.

The gear root fatigue crack usually initiates in the position where the root stress is maximum. Hence, the crack initiation position is assumed as the position with maximum gear root stress. Figure 8 shows the evolution of gear root stress as the distance from the gear root centre increases. The maximum gear root stress position is closer to the gear root centre as the rim thickness decreases. The distance between the gear root centre and the maximum root stress position reduces from 1.50 mm through 1.40 mm to 1.35 mm when the backup ratio decreases from 1 to 0.5, and then to 0.3.

**Figure 9:**
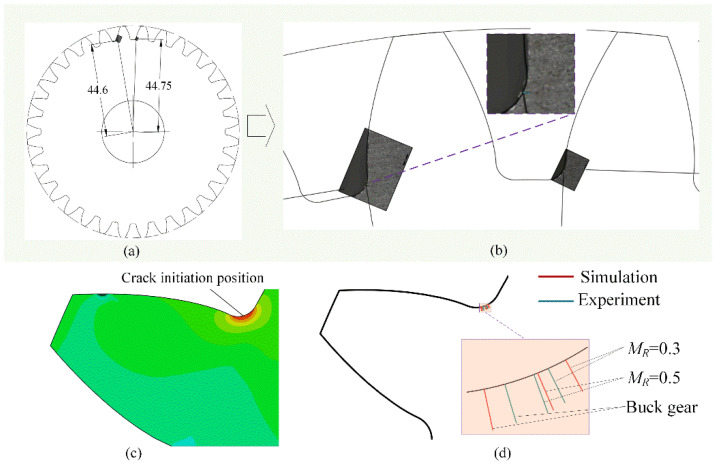
Comparison of crack initiation position between experiment (**a**,**b**) and simulation (**c**,**d**).

Figure 9 shows a comparison of crack initiation positions between the simulations and experiments. Specifically, Figure 9a,b shows the experimental results of gear crack initiation, in which the values of 44.6 mm and 44.75 mm represent the radius of the crack initiation position of the buck gear. Figure 9c shows the maximum gear root stress position in the FE simulation, namely the simulated gear root crack initiation position. Figure 9d shows that the simulated results for different backup ratios agree well with the experimental results.

### 4.3. Crack Propagation Path

**Figure 10:**
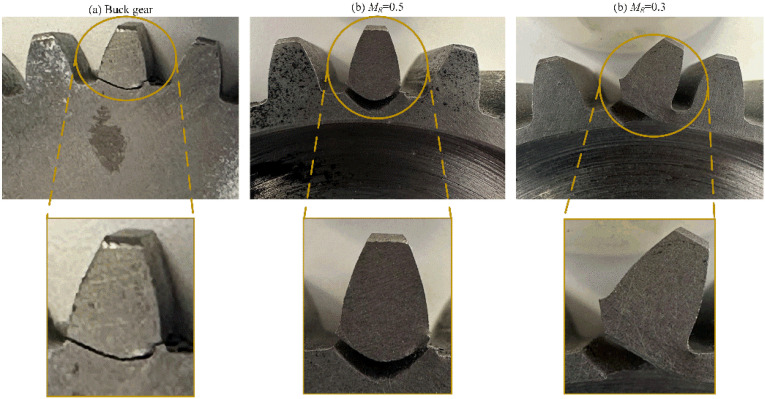
Examples of experimental results obtained from the three wheels.

**Figure 11:**
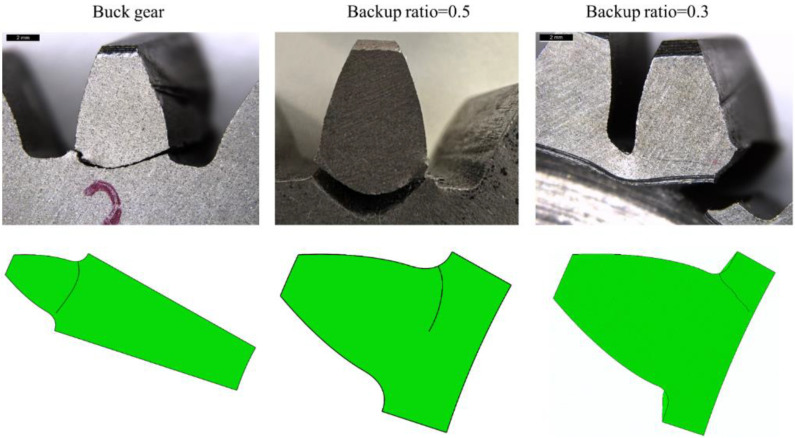
Comparison of gear root crack propagation between experiment and simulation.

The crack propagation path is influenced by the crack initiating position. In the experimental tests, the teeth were not notched; hence, the initiation point was not forced but is in a position given by the stress state, as explained above. Figure 10 shows the experimental results obtained from the three tested wheels. The buck gear and the gear with the backup ratio M_R_ = 0.5 show a safer crack propagation path (the cracks propagated through the tooth thickness), while a catastrophic failure (the crack propagated through the rim thickness) occurs in the wheel with the backup ratio M_R_ = 0.3. Figure 11 shows the comparison of the gear root crack propagation paths between the experiment and simulation for different backup ratio gears. The prefabricated initiated cracks in FE simulations located in different root surfaces for different backup ratio gears are shown in Figure 9d. The simulated crack propagation paths for different backup ratio gears agree well with the experimental tests.

**Figure 12:**
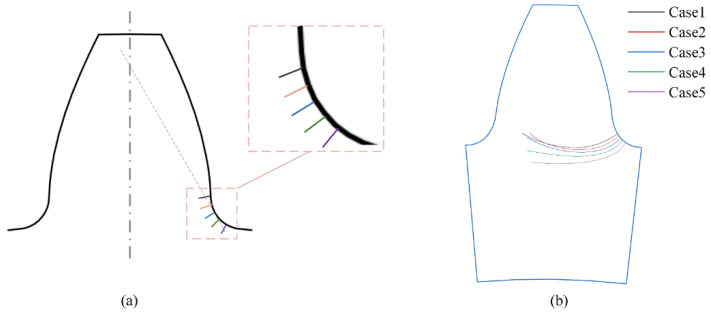
Gear crack propagation path for different crack initiation positions (**a**) the initial crack positions, (**b**) the gear root crack propagation paths.

To investigate the effect of crack initiation position on gear crack propagation path, different crack initiation position cases are considered in the numerical simulations. Table 4 lists the detailed configurations of the initial crack. The radius of the crack initiation position ranges from 44.80 mm to 44.40 mm. An initial crack of 0.2 mm is set in the gear root for each case, as shown in Figure 12. The results show that all the cracks propagate from one side of tooth root to another side in the backup ratio 1 gear despite different crack initiation positions. This indicates that the cracks will propagate along a similar path for the different cases.

## 5. Conclusions

In this work, the root crack propagation behaviour of thin-rim gears is investigated through experimental tests and numerical simulations. The crack initiation position and crack propagation path for different backup ratio gears are simulated through gear finite element (FE) models. The crack initiation position is determined using the maximum gear root stress position. The extended FE method is utilized to simulate the gear root crack propagation. The gear root crack simulation is verified by conducting gear experimental tests for different backup ratio gears based on a single tooth bending test device. The main conclusions can be drawn as follows:

(1) The gear bending stress increases dramatically as the rim thickness decreases. The effect of rim thickness on the gear bending stress needs to be considered when the back ratio is less than 0.5. The gear root crack initiation position is closer to the gear root centre as the rim thickness decreases. The distance between the gear root centre and the crack initiation position reduces from 1.50 mm through 1.40 mm to 1.35 mm when the backup ratio decreases from 1 through 0.5 to 0.3.
(2) When the gear backup ratio is larger than 0.5, the root crack propagates through the tooth thickness; yet, the crack in the wheel with a backup ratio of 0.3 propagates through the rim thickness and finally results in catastrophic failure.

## Figures and Tables

**Table 1:** Gear basic geometry parameters.

Teeth Number	*Z* = 32	Pressure Angle	$\alpha_{n}\;=\;20^{\circ }$
Module	*m_n_* = 3 mm	Gear tooth width	*B* = 20 mm
Shifting coefficient	*x_n_* = 0	Tooth addendum coefficients	1
Material	C45E		

**Table 2:** Chemical composition of C45E gear steel material.

C	Si	Mn	S	P	Cr	Ni	Mo	Al
0.44	0.22	0.63	0.004	0.01	0.31	0.03	0.04	0.022

**Table 3:** Gear bending stress (MPa).

Backup Ratios	ISO	Finite Element Simulation (Minimum Mesh Size)			
		0.2 mm	0.1 mm	0.05 mm	0.025 mm
0.3	365	562	683	698	708
0.5		381	428	449	458
1		328	395	415	429
Buck		356	388	413	430

**Table 4:** Different crack initiation positions for backup ratio 1 gear.

	Case 1	Case 2	Case 3	Case 4	Case 5
Radius of crack initiation position (mm)	44.80	44.70	44.60	44.50	44.40

## Data Availability

Not applicable.

## Nomenclature

$h_{\mathrm{FE}}$	the bending moment arm, mm
$s_{\mathrm{Fn}}$	the tooth root normal chord, mm
$t_{R}$	the rim thickness, mm
$t_{W}$	the web thickness, mm
$x_{n}$	the shifting coefficient
*B*	face width, mm
HPSTC	the highest point of single tooth contact
$M_{R}$	the gear backup ratio
$M_{W}$	the gear web ratio
$Y_{F}$	the geometry factor
$Y_{S}$	the stress concentration factor
Z	the teeth number
$F_{n}$	the normal loading force applied on the tooth at HPSTC, N
$F_{t}$	the tangential loading force applied on the tooth at HPSTC, N
$\alpha_{0}$	the normal $\mathrm{pressure}\;\mathrm{angle},\;\alpha_{0}=20^{\circ }$
$\alpha_{\mathrm{Fen}}$	the load direction angle
$\rho_{F}$	the radius of the root fillet, mm
